# Clinical Epidemiology of Cancer in People Living With HIV in Germany: Retrospective, Observational, Multicenter Federated Claims Data Analysis

**DOI:** 10.2196/81092

**Published:** 2026-02-13

**Authors:** Bastian Reiter, Stefanie Andreas, Linnea Schumann, Bernd Bender, Isabel Schnorr, Andreas Heidenreich, Christoph Stephan, Andrea Prunotto, Andrea Laukhuf, Julius Wehrle, Matthias Müller, Fabio Paul Aubele, Marlien Hagedorn, Fady Albashiti, Ulrich Seybold, Julia Roider, Melanie Stecher, Daniel Maier, Jörg Janne Vehreschild

**Affiliations:** 1 Institute for Digital Medicine and Clinical Data Sciences, Faculty of Medicine, Goethe University Frankfurt, Marienburgstrasse 6 Haus 97E / 2. OG, Frankfurt am Main, 60528, Germany, 49 15201483680; 2 Medical Department 2, Center for Internal Medicine, University Hospital Frankfurt, Frankfurt am Main, Germany; 3 German Cancer Consortium (DKTK), partner site Frankfurt/Mainz, a partnership between DKFZ and University Hospital Frankfurt, Frankfurt am Main, Germany; 4 Data Integration Center (DIC), University Hospital Frankfurt, Frankfurt am Main, Germany; 5 Medical HIV Treatment and Research Unit, HIVCenter, University Hospital Frankfurt, Frankfurt am Main, Germany; 6 Data Integration Center, Medical Center and Faculty of Medicine, University of Freiburg, Freiburg im Breisgau, Germany; 7 Department of Medicine I, Medical Center and Faculty of Medicine, University of Freiburg, Freiburg im Breisgau, Germany; 8 Department of Medicine II (Gastroenterology, Hepatology, Endocrinology and Infectious Diseases), Medical Center and Faculty of Medicine, University of Freiburg, Freiburg im Breisgau, Germany; 9 Department of Infection Medicine, Medical Service Centre Clotten, Freiburg im Breisgau, Germany; 10 Medical Data Integration Center, LMU University Hospital, LMU Munich, Munich, Germany; 11 Neu-Ulm University of Applied Sciences, Neu-Ulm, Germany; 12 Division of Infectious Diseases, LMU University Hospital, LMU Munich, Munich, Germany; 13 Department I of Internal Medicine, Faculty of Medicine and University Hospital Cologne, University of Cologne, Cologne, Germany; 14 German Center for Infection Research (DZIF), partner site Bonn-Cologne, Cologne, Germany

**Keywords:** secondary data analysis, federated analysis, real-world data, HIV, cancer, malignancy, people living with HIV and cancer, inpatient care, claims data, Section 21 German Hospital Fees Act

## Abstract

**Background:**

People living with HIV are at increased risk for developing cancer, a leading cause of death in this population. The management of cancer in people living with HIV is particularly challenging, necessitating specialized, interdisciplinary care. However, insights into cancer care provision for people living with HIV in Germany remain scarce.

**Objective:**

This study analyzed inpatient cancer care for people living with HIV, comparing treatment patterns and complications with those of an HIV-negative control group. Using claims data from 3 German university hospitals related to admissions between 2005 and 2022, we aimed to identify care disparities and provide evidence to support improved cancer management.

**Methods:**

A customized federated approach was used to analyze inpatient claims data of patients across the 3 data-holding institutions. The data included demographics, diagnoses, procedures, and treatment codes as well as discharge information. Using nearest-neighbor matching, we analyzed demographic features, cancer diagnoses, anticancer therapies, and outcomes in people living with HIV and cancer and for a control group of HIV-negative patients with cancer.

**Results:**

Among 162,380 patients, 907 (0.6%) were people living with HIV and cancer. The count of cancer diagnoses declined over time, particularly for AIDS-defining cancers (total cancer diagnoses: *P*=.001; AIDS-defining cancers: *P*=.002), with a shift toward older age at diagnosis. Compared with matched controls, people living with HIV and cancer had longer hospital stays, experienced more postchemotherapy complications (cancer with HIV: 64/907, 15.6%; cancer without HIV: 20/907, 5.5%; *P*<.001), and showed higher rates of metastasis after initial diagnosis (cancer with HIV: 128/267, 47.9%; cancer without HIV: 97/287, 33.8%; *P*<.001). People living with HIV and cancer also showed increased in-hospital mortality, although mortality declined over time (*P*=.02). Our data suggested differences in documented therapy modalities between the compared groups, with people living with HIV and cancer receiving more chemo- and immunotherapy and less surgery.

**Conclusions:**

Using federated analysis techniques, we were able to show that cancer diagnoses and mortality among people living with HIV in Germany have decreased over time; however, disparities in treatment and outcomes persisted as compared with HIV-negative patients with cancer. Our findings underscore the need for tailored, multidisciplinary care strategies to improve cancer management for this population.

## Introduction

Despite advances in treatment and prevention, HIV remains a major global health threat, with 39.9 million cases recorded worldwide in 2023 [1]. In Germany, HIV incidence has stabilized, and treated individuals no longer exhibit increased mortality, which is seen as a contributing factor to a rise in HIV prevalence [2]. The decline in AIDS-related deaths is largely attributed to effective antiretroviral therapy (ART) [3-6]. However, people living with HIV still face a significantly higher cancer risk, particularly for infection-related cancers. These occur approximately 30 times more frequently in female people living with HIV and up to 100 times more frequently in male people living with HIV as compared with the general population [7-10].

HIV-associated cancers in people living with HIV can be categorized as AIDS-defining (AD) and non–AIDS-defining (NAD), with AD cancers including Kaposi sarcoma, non-Hodgkin lymphoma, and invasive cervical cancer [11]. NAD cancers are further divided into virus-associated (virus-NAD) and non–virus-associated NAD types. Virus-NAD cancers linked to coinfections include lung, anal, vulvar, penile, oral, and pharyngeal cancers; Hodgkin lymphoma; and hepatocellular carcinoma [8,9,12,13].

Since the introduction of ART, the incidence of AD cancers has decreased, whereas the incidence of virus-NAD and non–virus-associated NAD cancers has increased [7,14,15]. Due to increased life expectancy, both virus-associated and non–virus-associated NAD cancers have become leading causes of death among people living with HIV, surpassing deaths directly attributable to HIV infection [5,16,17]. Although combination ART has improved survival and enables full-dose chemotherapy [18], managing cancer in people living with HIV remains complex. Immunosuppression, drug interactions with ART, and increased treatment toxicity pose significant challenges, requiring a multidisciplinary care approach involving oncology, hematology, infectious diseases, pharmacy, and supportive care specialists [19]. Given these complexities, people living with HIV and cancer may need to travel longer distances to access appropriate, specialized care.

While cancer epidemiology in people living with HIV is well-documented [7-10,12-15,19], evidence on cancer care in people living with HIV—particularly regarding treatment and outcomes—remains limited. Existing data, primarily from US registries, suggest that people living with HIV may receive cancer treatment less frequently than HIV-negative patients [20,21].

Using claims data from 3 German university hospitals, we analyzed inpatient cancer care for people living with HIV in Germany and compared it to that of an HIV-negative control group.

## Methods

### Study Data

This multicenter retrospective study used claims data collected under Section 21 of the German Hospital Fees Act (German: Krankenhausentgeltgesetz, KHEntgG). This law requires hospitals to report individual patient billing records to the federal institute for the hospital payment system (Institut für das Entgeltsystem im Krankenhaus). The data were sourced from data integration centers [22,23] of university hospitals in 3 large German cities (Frankfurt, Freiburg, and Munich).

The raw data included patient demographics and basic diagnostic- and treatment-related information (*International Statistical Classification of Diseases and Related Health Problems, Tenth Revision* [*ICD-10*]), as well as operation and procedure codes (Operationen- und Prozedurenschlüssel [OPS]; for more details, refer to Table S1 in Multimedia Appendix 1).

### Cohort Selection

Inclusion criteria required patients to be aged 18 years or older at the time of inpatient admission. The observation period was defined from January 1, 2005, to December 31, 2022. For inclusion, each patient also required at least 1 documented *ICD-10* code indicating either a cancer diagnosis or an HIV infection (Table S2 in Multimedia Appendix 1).

### Federated Data Analysis Approach

To analyze sensitive patient data distributed across multiple university hospitals, we used a federated approach; this means individual patient data were not transferred to a central computer to conduct pooled analysis. Instead, analysis scripts were sent to and executed locally at each participating hospital. Only aggregated or anonymized data were subsequently transferred to be jointly analyzed.

### Data Preprocessing and Patient Matching

A harmonized input data model (Table S3 in Multimedia Appendix 1) was developed to enable consistent and standardized analysis across study sites. Uniform value formats and restrictions were defined to ensure that only valid and consistent cases and data entries were included. A case was defined as a single hospitalization for a given patient. Using pseudonymized patient identifiers, individual hospital stays were linked to the same patient, allowing for multiple documented cases per patient during the observation period. Special focus was placed on plausibility checks to correctly classify main cancer and HIV diagnoses as well as therapeutic interventions (Tables S2, S4, and S5 in Multimedia Appendix 1). The data preprocessing resulted in a ready-for-analysis data model customized for the purpose of our study (Table S6 in Multimedia Appendix 1).

Included patients were categorized into 1 of 3 groups: cancer with HIV (cancer+/HIV+), cancer without HIV (cancer+/HIV–), and HIV without cancer (cancer–/HIV+) (Tables S2 and S5 in Multimedia Appendix 1). For patients with cancer, the first recorded cancer diagnosis was designated as the primary cancer diagnosis. For each patient group, frequencies of demographic and cancer-related, case-related, and therapy-related variables were summarized annually.

Patients in the cancer+/HIV+ group were matched with patients in the cancer+/HIV– group using 1:1 nearest-neighbor matching [24], based on age at cancer diagnosis, sex, year of cancer diagnosis, cancer topography group, presence of carcinoma in situ, and comorbidity score at the time of diagnosis (Table S7 in Multimedia Appendix 1). The comorbidity score was calculated using van Walraven weights for the Elixhauser comorbidity groups [25]. HIV infection and AIDS were assigned to a weight of zero, and cancer diagnoses beyond the primary diagnosis were considered comorbidities. Patient matching was performed to compare therapy-related data, including anticancer therapy frequencies, complications following chemotherapy (eg, transfer to the intensive care unit, dialysis, and mechanical ventilation), and outcomes (derived from the last recorded discharge reason) between the cancer+/HIV+ and cancer+/HIV– groups.

### Statistical Analysis

**Figure 1.**
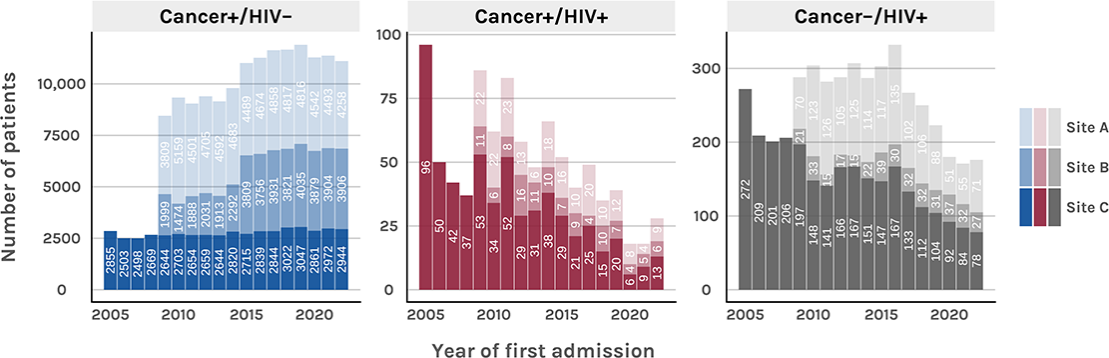
Number of patients in each defined group per year and per site (2005‐2022).

Relative frequencies of individual characteristics were calculated for each group across the entire study period (2005‐2022). For matched group comparisons, *P* values from the Fisher exact test were calculated. Trend analyses were based on data from 2009 to 2022, the period for which data were available from all 3 sites (Figure 1). Poisson regression models were used to assess temporal trends in age at cancer diagnosis and cancer-related diagnoses within the cancer+/HIV+ group. When overdispersion was detected, negative binomial regression models were used as an alternative [26].

For each regression model, the number of patients or diagnoses was used as the dependent variable, with the year of cancer diagnosis as the predictor. In contrast, for therapy-related trends, linear regression models were fitted using proportions as the dependent variable.

To assess travel distances for each patient group, the geographical distance between the centroid of each patient’s postal code area at the time of first admission and the hospital location was estimated using the Haversine formula [27], providing an approximation of how far patients in the 3 subgroups traveled to receive care. Additionally, the catchment areas of the individual sites were visualized by mapping the distribution of patients to 3-digit postal code areas for each group (Figure S1 in Multimedia Appendix 1).

All statistical analyses were conducted using R (version 4.3.2; R Foundation for Statistical Computing) [28]. Nearest-neighbor matching was performed with the MatchIt package [24]. The Fisher exact test as well as linear and Poisson regression models were computed with the stats package [28], while negative binomial regressions were modeled using the MASS package [29]. Travel distances for the patient subgroups were calculated with the geosphere [27] and sf [30] packages.

### Ethical Considerations

This retrospective study was conducted after consultation with the responsible ethics committees (research ethics committee, Faculty of Medicine, Goethe University Frankfurt; 274/18; research ethics committee, University of Freiburg: 22-1279-S1-retro). In accordance with national law, no informed consent was obtained since only pseudonymized patient data was used for analysis. Furthermore, the federated data analysis approach ensured data privacy through aggregation of individual-level data.

## Results

### Study Cohort

**Figure 2.**
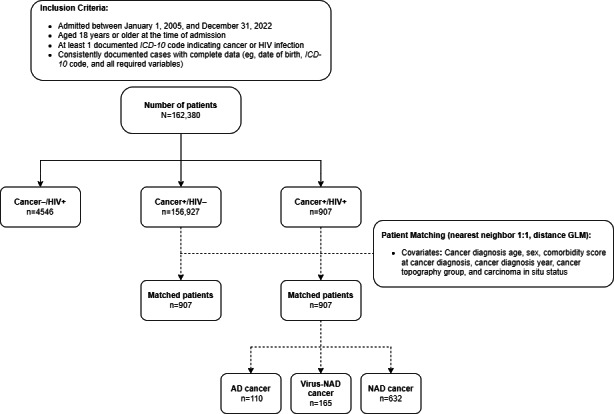
Inclusion, grouping, and matching of patients with the respective number of patients. AD: AIDS-defining; *ICD-10: International Statistical Classification of Diseases and Related Health Problems, Tenth Revision*; GLM: generalized linear model; NAD: non–AIDS-defining; Virus-NAD: cancer associated with HIV infection but not considered AIDS-defining.

Between 2005 and 2022, a total of 162,380 eligible inpatients were recorded across all 3 sites and patient groups (Figure 2). Among these, 907 (0.6%) patients were classified as people living with HIV and cancer (cancer+/HIV+), exhibiting at least 1 *ICD-10* code from both diagnosis groups. A total of 156,927 (96.8%) patients had at least 1 cancer-related *ICD-10* code without an HIV diagnosis (cancer+/HIV–), while 4546 (2.8%) patients were identified as people living with HIV without cancer (cancer–/HIV+). Figure 1 shows the annual distribution of patients in each group by site.

Patient demographics and admission-related characteristics for the 3 patient groups are displayed in Table 1. At the time of first admission, people living with HIV and cancer (cancer+/HIV+) were younger than HIV-negative patients with cancer (cancer+/HIV–), but older than people living with HIV without a cancer diagnosis (cancer–/HIV+). Fewer than one-quarter (214/907, 23.6%) of people living with HIV who have cancer were female.

**Table 1.** Demographic and admission-related characteristics of the 3 patient groups (2005-2022).

Characteristics	Cancer+/HIV– (n=156,927), n (%)	Cancer+/HIV+ (n=907), n (%)	Cancer–/HIV+ (n=4546), n (%)
Female patients	72,231 (46.0)	214 (23.6)	1441 (31.7)
Age (years) at first relevant (cancer or HIV associated) admission			
18‐39	13,373 (8.5)	176 (19.4)	1781 (39.2)
40‐59	44,984 (28.7)	536 (59.1)	2204 (48.5)
60‐79	80,335 (51.2)	192 (21.2)	547 (12.0)
>80	18,235 (11.6)	3 (0.3)	14 (0.3)
Admission count per patient			
1	58,444 (37.2)	144 (15.9)	2438 (53.6)
2‐4	62,840 (40.0)	351 (38.7)	1674 (36.8)
5‐10	27,877 (17.8)	301 (33.2)	356 (7.8)
More than 10	7766 (4.9)	111 (12.2)	78 (1.7)
Average length of stay per admission (days)			
Up to 7	77,096 (49.1)	307 (33.8)	2204 (48.5)
7‐14	49,898 (31.8)	298 (32.9)	1191 (26.2)
14‐30	23,194 (14.8)	233 (25.7)	835 (18.4)
More than 30	6739 (4.3)	69 (7.6)	316 (7.0)

On average, patients in the cancer+/HIV+ group exhibited the highest admission frequencies among the 3 groups: 2 to 4 admissions in 38.7% (351/907), 5 to 10 admissions in 33.2% (301/907), and >10 admissions in 12.2% (111/907) of all observed patient histories.

### Cancer Incidence and Cancer Types in People Living With HIV

Among 907 patients with both diagnoses (cancer+/HIV+), the data suggest that 504 (55.6%) patients received HIV and cancer diagnoses simultaneously or within a short time interval. In 332 (36.6%) patients, the HIV diagnosis preceded the cancer diagnosis, whereas in 71 (7.8%) patients, the first recorded cancer diagnosis was documented before the HIV diagnosis (Table S8 in Multimedia Appendix 1).

**Figure 3.**
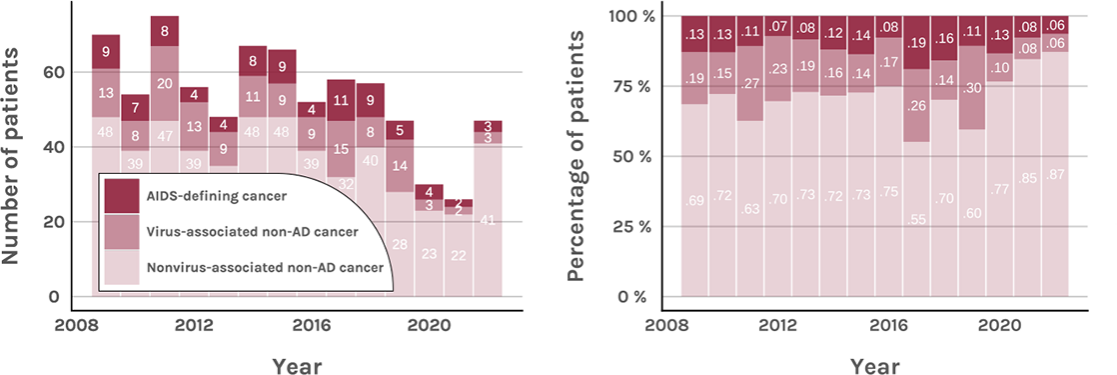
Absolute frequencies and proportions of cancer categories in people living with HIV and cancer (2009-2022). AD: AIDS defining.

While fluctuating over the observation period, the total number of cancer diagnoses in people living with HIV decreased significantly (*P*=.002). Among the cancer groups (AD, virus-NAD, and non–virus-associated NAD; Figure 3), the most notable decline was observed in the frequency of virus-associated NAD cancer diagnoses. In total, 110 (12.1%) diagnoses were classified as AD, 165 (18.2%) as virus-associated NAD, and 632 (69.7%) as non–virus-associated NAD. Among specific cancer subtypes, a significant reduction was observed in diagnoses involving lymphoid and hematopoietic tissues (*P*=.005), especially in non-Hodgkin lymphomas (*P*=.002) and nonfollicular lymphomas (*P*=.008).

**Figure 4.**
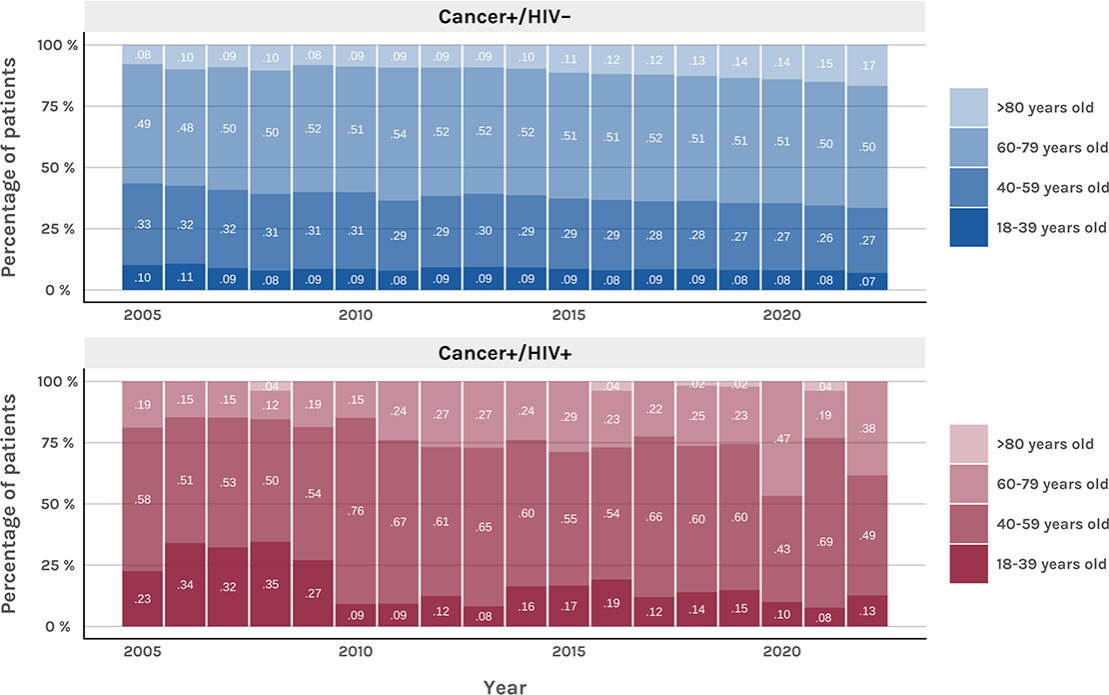
Proportions of grouped age at cancer diagnosis in people living with HIV and cancer and (unmatched) HIV-negative patients with cancer (2005-2022).

We observed an increase in the age at cancer diagnosis in both people living with HIV and HIV-negative patients with cancer (Figure 4). Consistent with this finding, regression analysis indicated a significant decrease in the proportion of patients younger than 60 years between 2009 and 2022 (*P*=.002).

**Figure 5.**
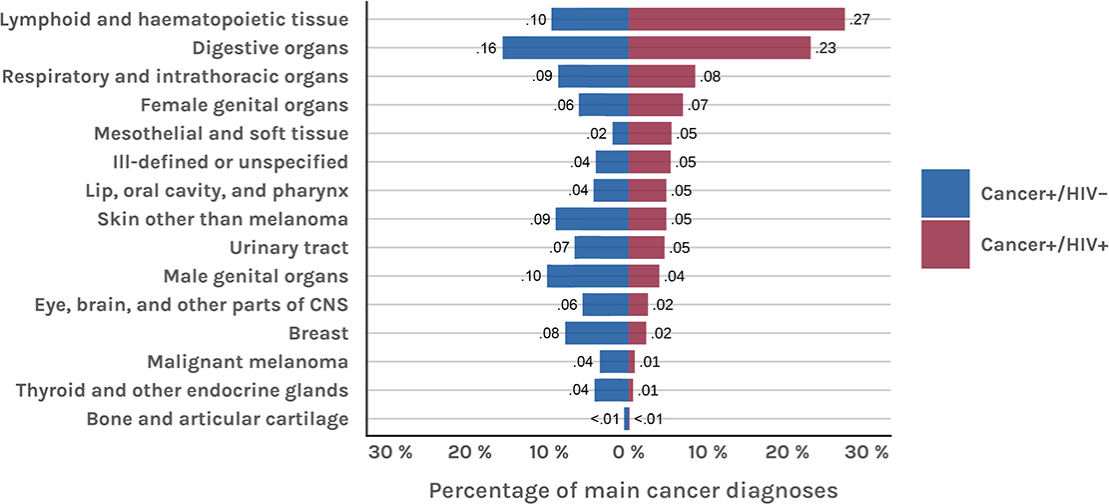
Most common cancer types in people living with HIV and cancer, sorted by frequency. CNS: central nervous system.

A comparison of cancer topography frequencies between people living with HIV and cancer (cancer+/HIV+) and HIV-negative patients with cancer (cancer+/HIV–) is presented in Figure 5.

In the matched comparison between cancer+/HIV+ and cancer+/HIV–, results showed an overall comparable prevalence of metastasis; however, metastasis was present at time of cancer diagnosis more often in the cancer+/HIV– group, whereas patients in the cancer+/HIV+ group seemed to develop metastasis after initial diagnosis more often than their HIV-negative matches (cancer+/HIV+: 128/267, 47.9%; cancer+/HIV–: 97/287, 33.8%; *P*<.001).

### Anticancer Treatment of People Living With HIV

People living with HIV and cancer (cancer+/HIV+) received anticancer therapies at rates comparable to those of HIV-negative patients with cancer (cancer+/HIV–) but were more frequently treated with chemotherapy and immunotherapy, whereas patients in the cancer+/HIV– group seemed to have received more stem cell therapy (Table 2). Our results show a significant decrease in the use of chemotherapy in the cancer+/HIV+ group between 2009 and 2022 (*P*=.02). People living with HIV and cancer presumably showed a higher rate of adverse events following chemotherapy than patients in the control group (cancer+/HIV+: 64/409, 15.6%; cancer+/HIV–: 20/361, 5.5%; *P*<.001). Additionally, the cancer+/HIV+ group was less frequently discharged home (516/713, 72.4%; *P*<.001) compared with the cancer+/HIV– group (583/714, 81.7%) and showed higher in-hospital mortality (cancer+/HIV+: 135/713, 18.9%; cancer+/HIV–: 90/714, 12.6%; *P*=.001).

**Table 2.** Comparison between people living with HIV and cancer and the matched patients with cancer without HIV.

Characteristics	Cancer+/HIV–, n (%)	Cancer+/HIV+, n (%)	*P* value^a^
Metastasis occurrence (n=907)			
Metastasis documented	287 (31.6)	267 (29.4)	.33
Metastasis at time of cancer diagnosis	190 (66.2)	139 (52.1)	<.001
Metastasis after cancer diagnosis	97 (33.8)	128 (47.9)	<.001
Therapy modalities (n=907)			
Any major therapy documented	631 (69.6)	639 (70.5)	.72
Surgery	285 (31.4)	251 (27.7)	.09
Chemotherapy	361 (39.8)	409 (45.1)	.03
Immunotherapy	90 (9.9)	138 (15.2)	<.001
Radiotherapy	158 (17.4)	169 (18.6)	.54
Stem cell therapy	84 (9.3)	33 (3.6)	<.001
Bone marrow transplant	12 (1.3)	0 (0.0)	.001
Complications after chemotherapy (n=907)			
Chemotherapy documented	361 (39.8)	409 (45.1)	.03
Complication after chemotherapy	20 (5.5)	64 (15.6)	<.001
Average length of stay per admission (days; n=714)^b^			
Up to 7	319 (44.7)	228 (31.9)	<.001
7-14	207 (29.0)	232 (32.5)	.17
14-30	133 (18.6)	198 (27.7)	<.001
More than 30	55 (7.7)	56 (7.8)	>.99
Last documented discharge category^c^			
Home	583 (81.7)	516 (72.4)	<.001
Deceased	90 (12.6)	135 (18.9)	.001
Other hospital	24 (3.4)	28 (3.9)	.58
Rehabilitation or residential care	11 (1.5)	20 (2.8)	.11
Hospice care	6 (0.8)	14 (2.0)	.08

a *P* values were calculated using the Fisher exact test.

b Data were available from only 2 sites (n=714 for both groups).

c Data were available from only 2 sites (n=714 for cancer+/HIV– and n=713 for cancer+/HIV+).

**Figure 6.**
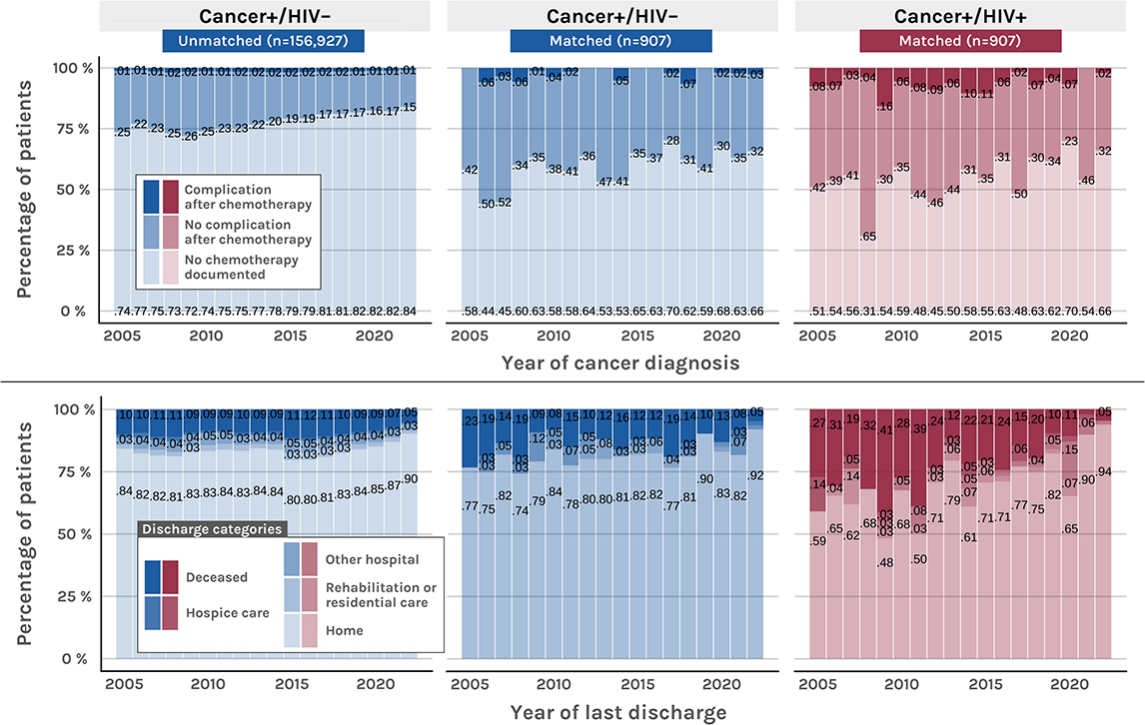
Comparison of complication rates after chemotherapy and last documented discharge categories between unmatched cancer+/HIV– and matched cancer+/HIV– and cancer+/HIV+.

Figure 6 depicts a comparison of temporal trends in complication rates after chemotherapy and discharge reasons across the total observed HIV-negative cancer population and the matched cancer groups.

For more detailed results, refer to Tables S7, S8, and S9 in Multimedia Appendix 1. A visual abstract of the study is presented in Multimedia Appendix 2.

## Discussion

### Principal Findings

In this study, we examined epidemiological as well as treatment- and outcome-related characteristics of inpatients affected by HIV and cancer in 3 selected German university hospitals.

The overall number of cancer diagnoses in the cancer+/HIV+ group decreased between 2009 and 2022, primarily driven by a decline in AD cancers. This reduction was largely attributable to fewer cases of non-Hodgkin lymphomas, particularly within the nonfollicular lymphoma subgroup. Previous studies examining earlier observation periods found that following the introduction of ART, diagnoses of AD cancers, which had predominated in the early HIV era, began to decline, whereas NAD cancers became increasingly common over time [14,15].

The most frequently observed topographical categories in our study are consistent with those reported in previous research [8,10]. Lymphoma was the most frequent cancer diagnosis among people living with HIV, followed by cancers of the lung and bronchi, and the anus and anal canal. Previous studies have shown that, among NAD cancers, lung and anal cancers represent the greatest disease burden in high-income countries [31], which is in line with our findings. No increase over time was observed for typical age-related cancers, such as prostate cancer or malignant melanoma of the skin. However, it remains unclear whether this reflects stable incidence rates or a shift toward outpatient care.

The results showed an increase in the age at cancer diagnosis among people living with HIV, with fewer cases occurring in younger adults (<60 years). This trend could be explained by the decline in AD cancer diagnoses and is consistent with findings from previous studies [32]. Despite age matching, people living with HIV were diagnosed with cancer at younger ages compared with HIV-negative controls. This observation reflects the need for targeted screening strategies and emphasizes the importance of preventive measures for cancers linked to modifiable risk factors, such as smoking in the case of lung cancer [19,31] and human papillomavirus infection in cervical cancer [33].

Despite similar overall cancer treatment rates, people living with HIV received chemotherapy and immunotherapy more frequently but underwent slightly fewer surgical interventions. This disparity may be explained by differences in cancer type, stage at diagnosis, and tumor operability, as well as HIV-related factors such as immunosuppression, elevated postoperative infection risk, and potential interactions between ART and anesthesia [32,34]. Deviating from previous findings [19], cancers in our observed cohort of people living with HIV were more often diagnosed at premetastatic stages, which contradicts the lower prevalence of surgical therapy in this group.

Our findings highlight the need for improved posttreatment care. The longer and more frequent hospitalizations observed among people living with HIV and cancer require further investigation to determine whether they stem from greater comorbidity burden or distinct clinical challenges associated with HIV infection.

Chemotherapy use during hospitalization declined among people living with HIV, suggesting a shift toward outpatient care [35,36]. To confirm this hypothesis, further analyses of outpatient care data are necessary. The study also showed a decrease in mortality among people living with HIV over time. Together, these trends suggest that cancer care and outcomes for people living with HIV have improved over the past 2 decades; however, additional research focusing on outpatient care is needed.

Contrary to our results, we initially hypothesized that people living with HIV and cancer would need to travel longer distances to access complex, multidisciplinary care. According to a recent study [37], HIV prevalence in Germany tends to be higher in urban compared with rural areas, which may explain shorter travel distances to urban university hospitals for people living with HIV in our study. Further research is needed to examine rural health care settings and to determine whether local care delivers comparable treatment outcomes or if traveling longer distances offers substantial benefits for specific patient groups like people living with HIV and cancer.

### Federated Data Analysis

The use of federated data analysis offered significant advantages, including improved data privacy by keeping sensitive patient information at its original site while enabling collaboration across multiple institutions. Nonetheless, challenges such as data heterogeneity, data inconsistencies, and technical limitations required repeated script adjustments for each center. This led to increased time and staffing costs, as well as minor discrepancies between site-specific results. Specialized federated analysis software such as DataSHIELD (DataSHIELD Research Project) [38] could mitigate these drawbacks but was not available in this study. All script modifications were recorded in a GitLab repository to maintain transparency and traceability.

### Limitations

A key limitation of this study is that the results cannot be generalized to the overall care of people living with HIV and cancer in Germany, as the analysis was based on data from 3 university hospitals representing the tertiary care sector. Further studies are needed to include nonuniversity hospitals and the outpatient sector.

Our study used large retrospective claims data, which the hospitals primarily documented for the purpose of billing services. Although data processing involved thorough plausibility checks, the risk that procedures or diagnoses were missing is high; furthermore, relevant information such as cancer staging and systemic therapy substances or dosage information was not part of the available data.

Sample size variations arose due to technical issues, leading to stratified analyses for the periods 2005 to 2008, 2009 to 2014, and 2015 to 2022 (Tables S6 and S7 in Multimedia Appendix 1). Other limitations include potential misclassification of disease onset and the inability to associate specific therapies with cancer types in patients with multiple diagnoses, both of which may have influenced the findings.

In our study, we chose to categorize malignancies as AD vs NAD. Different categorizations such as hematological vs solid tumors could have led to more nuanced insights. Further research should critically consider such decisions and potentially include multiple categorizations.

### Conclusions

Our study offers valuable insights into the inpatient care of people living with HIV and cancer in Germany, revealing shifts in cancer epidemiology and an aging patient population. Although the incidence of AD cancers has declined, people living with HIV continue to experience longer hospital stays and higher rates of posttreatment complications. Reduced mortality and inpatient chemotherapy use suggest that cancer care and outcomes for people living with HIV have improved over the past 2 decades; however, additional research focusing on outpatient care is needed.

The study also highlights the potential of federated data analysis for multicenter research, while emphasizing the need for standardized data collection to overcome technical challenges and data inconsistencies across sites.

## Supplementary material

10.2196/81092 Multimedia Appendix 1 Supplementary tables containing structural and semantic metadata as well as detailed study results.

10.2196/81092 Multimedia Appendix 2 Visual abstract.

## Abbreviations

AD: AIDS-defining
ART: antiretroviral therapy
*ICD-10*: *International Statistical Classification of Diseases and Related Health Problems, Tenth Revision*
KHEntgG: German Hospital Fees Act (German: Krankenhausentgeltgesetz)
NAD: non–AIDS-defining
OPS: operation and procedure classification system (German: Operationen- und Prozedurenschlüssel)
virus-NAD: cancer associated with HIV infection but not considered AIDS-defining
