# Supplementary material for: Clinical Epidemiology of Cancer in People Living With HIV in Germany: Retrospective, Observational, Multicenter Federated Claims Data Analysis
Source: JMIR Public Health Surveill. 2026 Feb 13;12:e81092. doi: 10.2196/81092 (PMC12904353; doi:10.2196/81092)

## Appendix

**Table S1**. Selected data elements from the data set corresponding to §21 German Hospital Fees Act [German: Krankenhausentgeltgesetz (KHEntgG)].

| **Attribute Name (german)** | **Attribute Description** | **Obligatory** | **Data Type** |
| --- | --- | --- | --- |
| **Table: Fall** | | | |
| KH-internes-Kennzeichen | Hospital-specific Case ID | yes | character |
| Geburtsjahr | Year of birth | yes | numeric |
| Geschlecht | Sex | yes | character |
| PLZ | Postal code | yes | character |
| Aufnahmedatum | Admission date | yes | date |
| Aufnahmeanlass | Admission cause | yes | character |
| Entlassungsdatum | Discharge date | yes | date |
| Entlassungsgrund | Discharge reason | yes | character |
| Alter-in-Jahren-am-Aufnahmetag | Age at admission date | no | numeric |
| Patientennummer | Hospital-specific Patient ID | yes | character |
| Verweildauer-Intensiv | Time spent in Intensive Care | yes | numeric |
| **Table: FAB** | | | |
| Fachabteilung | Specialty department | yes | character |
| FAB-Aufnahmedatum | Admission date at Specialty department | yes | date |
| FAB-Entlassungsdatum | Discharge date at Specialty department | yes | date |
| KennungIntensivbett | Whether stay at Specialty department was in Intensive Care | yes | character |
| **Table: ICD** | | | |
| Diagnoseart | Diagnosis type | yes | character |
| ICD-Version | ICD-10-GM Version | yes | numeric |
| ICD-Kode | ICD-10 Code | yes | character |
| Sekundär-Kode | Secondary ICD-10 Code | no | character |
| **Table: OPS** | | | |
| OPS-Version | OPS Version | yes | numeric |
| OPS-Kode | OPS Code | yes | character |
| OPS-Datum | OPS Date | yes | date |

**Table S2.** ICD-10 codes indicating malignancy or HIV infection.

| **ICD-10 Code** | **Interpretation** |
| --- | --- |
| **Cancer** | |
| C* | All malignant neoplasms (primary and secondary) |
| D0* | In-situ neoplasms |
| D37 - D48 | Neoplasms of uncertain / unknown behaviour |
| Z08 | Follow-up examination after treatment for malignant neoplasms |
| Z85, Z92.6 | Personal history of cancer / cancer therapy |
| **HIV** | |
| B20 | Human immunodeficiency virus [HIV] disease resulting in infectious and parasitic diseases |
| B21 | Human immunodeficiency virus [HIV] disease resulting in malignant neoplasms |
| B22 | Human immunodeficiency virus [HIV] disease resulting in other specified diseases |
| B23.x | Human immunodeficiency virus [HIV] disease resulting in other conditions |
| B24 | Unspecified human immunodeficiency virus [HIV] disease |
| Z21 | Asymptomatic human immunodeficiency virus [HIV] infection status |
| O98.7 | Human immunodeficiency virus [HIV] disease complicating pregnancy, childbirth and the puerperium |
| U60.x | Clinical category of HIV disease |
| U61.x | Count of CD4+-T-cells per microliter blood |
| U85 | Human immunodeficiency virus with resistance against virostatics |

*Table Notes*. Patients with at least one ICD-10 code for both cancer and HIV infection were assigned to the group “Cancer+/HIV+”. Patients with at least one ICD-10 code for cancer only were assigned to the patient group “Cancer+/HIV-“; and patients with at least one ICD-10 code for HIV only were assigned to the group “Cancer-/HIV+”.

**Table S3.** Harmonized Input Data Model used in federated processing of raw data.

| **Attribute Name** | **Data Type** | **Format** | **Value Restrictions** |
| --- | --- | --- | --- |
| **Table: Cases** | | | |
| PatientPseudonym | character |  |  |
| CasePseudonym | character |  |  |
| YearOfBirth | integer | 9999 | [1890 - 2006] |
| Sex | factor | c | {w, m, u, d, x} |
| PostalCode | character | 99999 |  |
| AdmissionDate | date | YYYY-mm-dd | [2005-01-01 - 2022-12-31] |
| AdmissionAge | integer |  | [18 - 120] |
| DischargeDate | date | YYYY-mm-dd | [2005-01-01 - 2022-12-31] |
| DischargeReason | factor | 999 | {01x, 02x, 03x, 04x, 059, 06x, 079, 089, 09x, 10x, 11x, 139, 14x, 15x, 179, 2x9, 309} |
| **Table: ICD10Codes** | | | |
| CasePseudonym | character |  |  |
| DiagnosisType | factor | CC | {HD, ND} |
| ICDVersion | integer | 9999 | [1990 - 2023] |
| ICDCode | character | C99.x |  |
| SecondaryICDCode | character | C99.x |  |
| **Table: OPSCodes** | | | |
| CasePseudonym | character |  |  |
| OPSVersion | integer | 9999 | [1990 - 2023] |
| OPSCode | character | 9999xx |  |
| OPSDate | date | YYYY-mm-dd | [2005-01-01 - 2022-12-31] |

**Table S4.** OPS codes indicating therapeutic procedure and used to detect potential adverse events or complications.

| **OPS Code** | **Interpretation** |
| --- | --- |
| **Therapy** |  |
| Starting with "5" but not with "5411" or "5936" | Surgery |
| Starting with "854" but not with "8547" or "8548" | Chemotherapy |
| Starting with "8547" | Immunotherapy |
| Starting with "852" | Radiotherapy |
| Starting with "853" | Nuclear medicine therapy |
| Starting with "8805" | Stem cell therapy |
| Starting with "5411" | Bone marrow transplant |
| Starting with "5936" | Potential CAR T-cell therapy |
| Starting with "8548" | Antiretroviral therapy |
| **Potential adverse event or complication** |  |
| Starting with one of "8701", "8704", "8706", "8712", "8713", "8714" | Mechanical ventilation |
| Starting with one of "8853", "8854", "8855", "8857" | Dialysis |

**Table S5.** ICD-10 codes used (in conjunction with secondary ICD10 codes) for classification of cancer categories: AIDS-defining (AD), HIV-associated non-AIDS-defining (virus-NAD), and non-AIDS-defining (NAD).

| **Cancer group** | **ICD10-Code** | **Cancer type** |
| --- | --- | --- |
| **AIDS-defining (AD)** | C46.* | Kaposi sarcoma |
|  | C53.* | Invasive cervical cancer |
|  | C82.* | Primary lymphoma / Follicular lymphoma |
|  | C83.* | Primary lymphoma / Non-follicular lymphoma |
|  | C84.* | Primary lymphoma / Mature T/NK-cell lymphoma |
|  | C85.* | Primary lymphoma / Unspecified Non-Hodgkin lymphoma |
|  | C86.* | Primary lymphoma / Other specified T/NK-cell lymphoma |
| **HIV-associated non-AIDS-defining (virus-NAD)** | C01 | Malignant neoplasm of base of tongue |
|  | C02.4 | Malignant neoplasm: Lingual tonsil |
|  | C09.* | Malignant neoplasm of tonsil, excl. tongue and pharynx |
|  | C10.* | Malignant neoplasm of oropharynx |
|  | C14.0 | Malignant neoplasm: Pharynx, unspecified |
|  | C14.2 | Malignant neoplasm: Waldeyer ring |
|  | C20 | Malignant neoplasm of rectum |
|  | C21.* | Malignant neoplasm of anus and anal canal |
|  | C22.0 | Liver cell carcinoma |
|  | C51.* | Malignant neoplasm of vulva |
|  | C52 | Malignant neoplasm of vagina |
|  | C60.* | Malignant neoplasm of penis |
|  | C81.* | Hodgkin lymphoma |
| **non-AIDS-defining (NAD)** | Neither code for AD nor virus-NAD | |

**Table S6.** Processed Data Model used for federated data analysis.

| **Attribute Name** | **Data Type** | **Format** | **Value Restrictions** |
| --- | --- | --- | --- |
| **Table: Diagnosis** | | | |
| PatientPseudonym | character |  |  |
| CasePseudonym | character |  |  |
| AdmissionDate | date | YYYY-mm-dd | [2005-01-01 - 2022-12-31] |
| AdmissionYear | integer | YYYY | [2005 - 2022] |
| AdmissionAge | integer |  | [18 - 120] |
| DischargeDate | date | YYYY-mm-dd | [2005-01-01 - 2022-12-31] |
| DischargeReason | factor | 999 | {01x, 02x, 03x, 04x, 059, 06x, 079, 089, 09x, 10x, 11x, 139, 14x, 15x, 179, 2x9, 309} |
| DischargeGroup | character |  |  |
| LengthOfStay | integer |  | > 0 |
| PostalCode | character | 99999 |  |
| DiagnosisType | factor | CC | {'HD', 'ND'} |
| ICDCodeShort | character | C99 |  |
| DiagnosisGeneral | character |  |  |
| ICDCode | character | C99.x |  |
| SecondaryICDCode | character | C99.x |  |
| DiagnosisDetail | character |  |  |
| HIVDiseaseGerman | character |  |  |
| HIVDiseaseClass | factor |  | {'Cancer', 'HIV proprietary', 'Infection'} |
| HIVInformationClass | factor |  | {'CD4+ count', 'HIV Stadium'} |
| HIVInformationValue | character |  |  |
| HIVCodingPlausibility | factor |  | {'Implausible', 'Plausible'} |
| HIVStatusInterpretation | factor |  | {'AIDS', 'HIV positive'} |
| HIVAssociation | factor |  | {'AIDS defining', 'HIV associated'} |
| IsCancerCode | logical |  | {TRUE, FALSE} |
| IsPotentialMainCancer | logical |  | {TRUE, FALSE} |
| IsPresumedMainCancerDiagnosis | logical |  | {TRUE, FALSE} |
| IsMetastasisCode | logical |  | {TRUE, FALSE} |
| IsPresumedMetastasisDiagnosis | logical |  | {TRUE, FALSE} |
| IsHIVCode | logical |  | {TRUE, FALSE} |
| IsPresumedHIVDiagnosis | logical |  | {TRUE, FALSE} |
| IsAIDSCode | logical |  | {TRUE, FALSE} |
| IsPresumedAIDSDiagnosis | logical |  | {TRUE, FALSE} |
| IsADCode | logical |  | {TRUE, FALSE} |
| IsADCodeCancer | logical |  | {TRUE, FALSE} |
| IsADCodeNonCancerous | logical |  | {TRUE, FALSE} |
| IsHIVNonADCodeCancer | logical |  | {TRUE, FALSE} |
| **Table: Patients** | | | |
| PatientPseudonym | character |  |  |
| PatientSubgroup | character |  | {'Cancer+/HIV-', 'Cancer+/HIV+', 'Cancer-/HIV+'} |
| YearOfBirth | integer | 9999 | [1890 - 2006] |
| Sex | factor | c | {w, m, u, d, x} |
| PrimaryPostalCode | character | 99999 |  |
| PatIsCancerCoded | logical |  | {TRUE, FALSE} |
| PatIsMetastasisCoded | logical |  | {TRUE, FALSE} |
| PatIsHIVCoded | logical |  | {TRUE, FALSE} |
| PatIsAIDSCoded | logical |  | {TRUE, FALSE} |
| CaseCount | integer |  | > 0 |
| MeanLengthOfStay | double |  | > 0 |
| DistinctCodeCount | integer |  | > 0 |
| DistinctCodeCount MainCancer | integer |  | >= 0 |
| DistinctCodeCountHIV | integer |  | >= 0 |
| DistinctCodeCountAIDS | integer |  | >= 0 |
| PresumedMainCancer DiagnosisDate | date |  |  |
| PresumedMetastasis DiagnosisDate | date |  |  |
| PresumedHIVDiagnosisDate | date |  |  |
| PresumedAIDSDiagnosisDate | date |  |  |
| FirstMainAdmissionDate | date |  | [2005-01-01 - 2022-12-31] |
| FirstMainAdmissionAge | integer |  | [18 - 120] |
| LastRecordedDischargeDate | date | YYYY-mm-dd | [2005-01-01 - 2022-12-31] |
| LastRecordedDischargeReason | factor | 999 | {01x, 02x, 03x, 04x, 059, 06x, 079, 089, 09x, 10x, 11x, 139, 14x, 15x, 179, 2x9, 309} |
| FirstMainAdmissionYear | integer | 9999 | [2005 - 2022] |
| MainRecordedTimeSpan | integer |  |  |
| TimeHIVToCancer | integer |  |  |
| TimeHIVToAIDS | integer |  |  |
| TimeAIDSToCancer | integer |  |  |
| TimeCancerToMetastasis | integer |  |  |
| ComorbidityScore | integer |  |  |

**Table S7**. Post-matching standardized mean differences for all matching variables across study sites.

| **Matching Variable** | **SMD (Site A)** | **SMD (Site B)** | **SMD (Site C)** |
| --- | --- | --- | --- |
| Age at main cancer diagnosis | 0,01 | 0,01 | 0,06 |
| Sex female | * | 0,08 | 0,03 |
| Comorbidity score at cancer diagnosis | * | 0,03 | 0,04 |
| Year of main cancer diagnosis | * | 0,04 | 0,08 |
| Main cancer is carcinoma in situ | 0,04 | 0,04 | 0,02 |
| **Cancer topography group** | | | |
| Bone and articular cartilage | 0 | 0 | 0 |
| Breast | 0 | 0,05 | 0,05 |
| Digestive organs | 0 | 0,07 | 0,10 |
| Eye, brain and other parts of CNS | 0 | 0,12 | 0,03 |
| Female genital organs | 0,04 | 0,10 | 0,02 |
| Ill-defined or unspecified | 0 | 0,10 | 0,03 |
| Lip, oral cavity, pharynx | 0,02 | 0,13 | 0,06 |
| Lymphoid and haematopoietic tissue | 0,01 | 0,14 | 0,01 |
| Male genital organs | 0,08 | 0,13 | 0,06 |
| Malignant melanoma | 0,05 | 0,13 | 0,02 |
| Mesothelial and soft tissue | 0,03 | 0,19 | 0,06 |
| Respiratory and intrathoracic organs | 0,02 | 0,00 | 0,04 |
| Skin other than melanoma | 0,07 | 0,16 | 0,06 |
| Thyroid and other endocrine glands | 0,06 | 0,09 | 0,06 |
| Urinary tract | 0,03 | 0,04 | 0,01 |

Abbreviations: SMD, Standardized Mean Difference; CNS, central nervous system
* SMD could not be calculated from aggregated site data

**Table S8.** Observed cancer categories in PLWH and unmatched HIV-negative population; Presumed order of HIV and cancer diagnosis.

| **Characteristics** | | **Cancer+/HIV-, n (%)** | **Cancer+/HIV+, n (%)** |
| --- | --- | --- | --- |
| **Observed cancer categories** | |  |  |
| 2005 - 2008 | AIDS-defining cancer | 187 (1.8%) | 23 (14.9%) |
|  | Virus-associated non-AD cancer | 857 (8.1%) | 28 (18.2%) |
|  | Non-virus-associated non-AD cancer | 9,481 (90.1%) | 103 (66.9%) |
| 2009 - 2014 | AIDS-defining cancer | 1,230 (2.2%) | 40 (10.8%) |
|  | Virus-associated non-AD cancer | 4,244 (7.7%) | 74 (20.0%) |
|  | Non-virus-associated non-AD cancer | 49,696 (90.1%) | 256 (69.2%) |
| 2015 - 2022 | AIDS-defining cancer | 1,806 (2.0%) | 47 (12.3%) |
|  | Virus-associated non-AD cancer | 5,141 (5.6%) | 63 (16.4%) |
|  | Non-virus-associated non-AD cancer | 84,285 (92.4%) | 273 (71.3%) |
| Total | AIDS-defining cancer | 3,223 (2.1%) | 110 (12.1%) |
|  | Virus-associated non-AD cancer | 10,242 (6.5%) | 165 (18.2%) |
|  | Non-virus-associated non-AD cancer | 143,462 (91.4%) | 632 (69.7%) |
| **Order of diagnosis** | |  |  |
| 2005 - 2008 | Cancer before HIV |  | 6 (3.9%) |
|  | Diagnosed simultaneously |  | 107 (69.5%) |
|  | HIV before cancer |  | 41 (26.6%) |
| 2009 - 2014 | Cancer before HIV |  | 32 (8.6%) |
|  | Diagnosed simultaneously |  | 217 (58.6%) |
|  | HIV before cancer |  | 121 (32.7%) |
| 2015 - 2022 | Cancer before HIV |  | 33 (8.6%) |
|  | Diagnosed simultaneously |  | 180 (47.0%) |
|  | HIV before cancer |  | 170 (44.4%) |
| Total | Cancer before HIV |  | 71 (7.8%) |
|  | Diagnosed simultaneously |  | 504 (55.6%) |
|  | HIV before cancer |  | 332 (36.6%) |

**Table S9. Time-stratified results of demographic and case-related information on the three patient groups**

| **Characteristics** | | **Cancer+/HIV-, n (%)** | | **Cancer+/HIV+, n (%)** | | **Cancer-/HIV+, n (%)** |
| --- | --- | --- | --- | --- | --- | --- |
| **Number of patients** | |  |  | |  | |
| 2005 - 2008 |  | 10,525 | 225 | | 888 | |
| 2009 - 2014 |  | 55,17 | 403 | | 1,756 | |
| 2015 - 2022 |  | 91,232 | 279 | | 1,902 | |
| Total |  | 156,927 | 907 | | 4,546 | |
| **Female patients** | |  |  | |  | |
| 2005 - 2008 |  | 4,901 (46.6%) | 60 (26.7%) | | 309 (34.8%) | |
| 2009 - 2014 |  | 25,618 (46.4%) | 82 (20.3%) | | 565 (32.2%) | |
| 2015 - 2022 |  | 41,712 (45.7%) | 72 (25.8%) | | 567 (29.8%) | |
| Total |  | 72,231 (46.0%) | 214 (23.6%) | | 1,441 (31.7%) | |
| **Age at first relevant admission** | |  |  | |  | |
| 2005 - 2008 | 18 - 39 years old | 1,003 (9.5%) | 64 (28.4%) | | 400 (45.0%) | |
|  | 40 - 59 years old | 3,372 (32.0%) | 122 (54.2%) | | 428 (48.2%) | |
|  | 60 - 79 years old | 5,172 (49.1%) | 39 (17.3%) | | 60 (6.8%) | |
|  | > 80 years old | 978 (9.3%) | 0 | | 0 | |
| 2009 - 2014 | 18 - 39 years old | 4,900 (8.9%) | 60 (14.9%) | | 704 (40.1%) | |
|  | 40 - 59 years old | 16,500 (29.9%) | 260 (64.5%) | | 856 (48.7%) | |
|  | 60 - 79 years old | 28,747 (52.1%) | 83 (20.6%) | | 192 (10.9%) | |
|  | > 80 years old | 5,023 (9.1%) | 0 | | ≤ 5 | |
| 2015 - 2022 | 18 - 39 years old | 7,470 (8.2%) | 52 (18.6%) | | 677 (35.6%) | |
|  | 40 - 59 years old | 25,112 (27.5%) | 154 (55.2%) | | 920 (48.4%) | |
|  | 60 - 79 years old | 46,416 (50.9%) | 70 (25.1%) | | 295 (15.5%) | |
|  | > 80 years old | 12,234 (13.4%) | ≤ 5 | | 10 (0.5%) | |
| Total | 18 - 39 years old | 13,373 (8.5%) | 176 (19.4%) | | 1,781 (39.2%) | |
|  | 40 - 59 years old | 44,984 (28.7%) | 536 (59.1%) | | 2,204 (48.5%) | |
|  | 60 - 79 years old | 80,335 (51.2%) | 192 (21.2%) | | 547 (12.0%) | |
|  | > 80 years old | 18,235 (11.6%) | ≤ 5 | | 14 (1.5%) | |
| **Admission count per patient** | |  |  | |  | |
| 2005 - 2008 | 1 admission | 4,335 (41.2%) | 24 (10.7%) | | 420 (47.3%) | |
|  | 2 - 4 admissions | 3,976 (37.8%) | 77 (34.2%) | | 348 (39.2%) | |
|  | 5 - 10 admissions | 1,726 (16.4%) | 81 (36.0%) | | 91 (10.2%) | |
|  | More than 10 admissions | 488 (4.6%) | 43 (19.1%) | | 29 (3.3%) | |
| 2009 - 2014 | 1 admission | 17,447 (31.6%) | 52 (12.9%) | | 826 (47.0%) | |
|  | 2 - 4 admissions | 21,862 (39.6%) | 153 (38.0%) | | 711 (40.5%) | |
|  | 5 - 10 admissions | 11,869 (21.5%) | 149 (37.0%) | | 182 (10.4%) | |
|  | More than 10 admissions | 3,992 (7.2%) | 49 (12.2%) | | 37 (2.1%) | |
| 2015 - 2022 | 1 admission | 36,662 (40.2%) | 68 (24.4%) | | 1,192 (62.7%) | |
|  | 2 - 4 admissions | 37,002 (40.6%) | 121 (43.4%) | | 615 (32.3%) | |
|  | 5 - 10 admissions | 14,282 (15.7%) | 71 (25.4%) | | 83 (4.4%) | |
|  | More than 10 admissions | 3,286 (3.6%) | 19 (6.8%) | | 12 (0.6%) | |
| Total | 1 admission | 58,444 (37.2%) | 144 (15.9%) | | 2,438 (53.6%) | |
|  | 2 - 4 admissions | 62,840 (40.0%) | 351 (38.7%) | | 1,674 (36.8%) | |
|  | 5 - 10 admissions | 27,877 (17.8%) | 301 (33.2%) | | 356 (7.8%) | |
|  | More than 10 admissions | 7,766 (4.9%) | 111 (12.2%) | | 78 (1.7%) | |
| **Average length of stay** | |  |  | |  | |
| 2005 - 2008 | Up to 7 days on avg. | 4,778 (45.4%) | 65 (28.9%) | | 362 (40.8%) | |
|  | 7 - 14 days on avg. | 3,601 (34.2%) | 77 (34.2%) | | 282 (31.8%) | |
|  | 14 - 30 days on avg. | 1,725 (16.4%) | 68 (30.2%) | | 177 (19.9%) | |
|  | > 30 days on avg. | 421 (4.0%) | 15 (6.7%) | | 67 (7.5%) | |
| 2009 - 2014 | Up to 7 days on avg. | 25,610 (46.4%) | 140 (34.7%) | | 866 (49.3%) | |
|  | 7 - 14 days on avg. | 18,269 (33.1%) | 129 (32.0%) | | 477 (27.2%) | |
|  | 14 - 30 days on avg. | 8,717 (15.8%) | 98 (24.3%) | | 298 (17.0%) | |
|  | > 30 days on avg. | 2,574 (4.7%) | 36 (8.9%) | | 115 (6.5%) | |
| 2015 - 2022 | Up to 7 days on avg. | 46,708 (51.2%) | 102 (36.6%) | | 976 (51.3%) | |
|  | 7 - 14 days on avg. | 28,028 (30.7%) | 92 (33.0%) | | 432 (22.7%) | |
|  | 14 - 30 days on avg. | 12,752 (14.0%) | 67 (24.0%) | | 360 (18.9%) | |
|  | > 30 days on avg. | 3,744 (4.1%) | 18 (6.5%) | | 134 (7.0%) | |
| Total | Up to 7 days on avg. | 77,096 (49.1%) | 307 (33.8%) | | 2,204 (48.5%) | |
|  | 7 - 14 days on avg. | 49,898 (31.8%) | 298 (32.9%) | | 1,191 (26.2%) | |
|  | 14 - 30 days on avg. | 23,194 (14.8%) | 233 (25.7%) | | 835 (18.4%) | |
|  | > 30 days on avg. | 6,739 (4.3%) | 69 (7.6%) | | 316 (7.0%) | |
| **Number of distinct cancer diagnoses** | |  |  | |  | |
| 2005 - 2008 | 1 distinct cancer | 7,671 (72.9%) | 145 (64.4%) | | - | |
|  | 2 distinct cancers | 2,029 (19.3%) | 46 (20.4%) | | - | |
|  | 3 or more distinct cancers | 825 (7.8%) | 34 (15.1%) | | - | |
|  | 1 distinct cancer | 36,962 (67.0%) | 259 (64.3%) | | - | |
| 2009 - 2014 | 2 distinct cancers | 11,720 (21.2%) | 99 (24.6%) | | - | |
|  | 3 or more distinct cancers | 6,488 (11.8%) | 45 (11.2%) | | - | |
|  | 1 distinct cancer | 63,539 (69.6%) | 205 (73.5%) | | - | |
|  | 2 distinct cancers | 19,905 (21.8%) | 52 (18.6%) | | - | |
| 2015 - 2022 | 3 or more distinct cancers | 7,788 (8.5%) | 22 (7.9%) | | - | |
|  | 1 distinct cancer | 108,172 (68.9%) | 609 (67.1%) | | - | |
|  | 2 distinct cancers | 33,654 (21.4%) | 197 (21.7%) | | - | |
|  | 3 or more distinct cancers | 15,101 (9.6%) | 101 (11.1%) | | - | |
| **Main cancer is carcinoma in situ** | |  |  | |  | |
| 2005 - 2008 |  | 338 (3.2%) | 24 (15.6%) | | - | |
| 2009 - 2014 |  | 1,747 (3.2%) | 25 (6.8%) | | - | |
| 2015 - 2022 |  | 2,776 (3.0%) | 23 (6.0%) | | - | |
| **Total** |  | 4,861 (3.1%) | 72 (7.9%) | | - | |
| **Cancer topography** | |  |  | |  | |
| **2005 - 2008** | Bone and articular cartilage | 47 (0.4%) | 0 | | - | |
|  | Breast | 1,146 (10.9%) | ≤ 5 | | - | |
|  | Digestive organs | 1,573 (14.9%) | 25 (16.2%) | | - | |
|  | Eye, brain and other parts of CNS | 752 (7.1%) | ≤ 5 | | - | |
|  | Female genital organs | 537 (5.1%) | 18 (11.7%) | | - | |
|  | Ill-defined or unspecified | 213 (2.0%) | 11 (7.1%) | | - | |
|  | Lip, oral cavity, pharynx | 780 (7.4%) | ≤ 5 | | - | |
|  | Lymphoid and haematopoietic tissue | 1,196 (11.4%) | 44 (28.6%) | | - | |
|  | Male genital organs | 414 (3.9%) | ≤ 5 | | - | |
|  | Malignant melanoma | 614 (5.8%) | 0 | | - | |
|  | Mesothelial and soft tissue | 123 (1.2%) | 14 (9.1%) | | - | |
|  | Respiratory and intrathoracic organs | 834 (7.9%) | 16 (10.4%) | | - | |
|  | Skin other than melanoma | 1,400 (13.3%) | ≤ 5 | | - | |
|  | Thyroid and other endocrine glands | 313 (3.0%) | 0 | | - | |
|  | Urinary tract | 583 (5.5%) | 9 (5.8%) | | - | |
| **2009 - 2014** | Bone and articular cartilage | 338 (0.6%) | ≤ 5 | | - | |
|  | Breast | 4,541 (8.2%) | 11 (3.0%) | | - | |
|  | Digestive organs | 8,736 (15.8%) | 81 (21.9%) | | - | |
|  | Eye, brain and other parts of CNS | 3,535 (6.4%) | 10 (2.7%) | | - | |
|  | Female genital organs | 3,613 (6.5%) | 15 (4.1%) | | - | |
|  | Ill-defined or unspecified | 2,082 (3.8%) | 20 (5.4%) | | - | |
|  | Lip, oral cavity, pharynx | 2,603 (4.7%) | 21 (5.7%) | | - | |
|  | Lymphoid and haematopoietic tissue | 5,804 (10.5%) | 111 (30.0%) | | - | |
|  | Male genital organs | 4,832 (8.8%) | 12 (3.2%) | | - | |
|  | Malignant melanoma | 1,634 (3.0%) | ≤ 5 | | - | |
|  | Mesothelial and soft tissue | 1,183 (2.1%) | 18 (4.9%) | | - | |
|  | Respiratory and intrathoracic organs | 4,870 (8.8%) | 26 (7.0%) | | - | |
|  | Skin other than melanoma | 4,535 (8.2%) | 21 (5.7%) | | - | |
|  | Thyroid and other endocrine glands | 2,818 (5.1%) | ≤ 5 | | - | |
|  | Urinary tract | 4,046 (7.3%) | 19 (5.1%) | | - | |
| **2015 - 2022** | Bone and articular cartilage | 528 (0.6%) | 0 | | - | |
|  | Breast | 6,868 (7.5%) | 6 (1.6%) | | - | |
|  | Digestive organs | 14,602 (16.0%) | 102 (26.6%) | | - | |
|  | Eye, brain and other parts of CNS | 4,828 (5.3%) | 8 (2.1%) | | - | |
|  | Female genital organs | 5,708 (6.3%) | 29 (7.6%) | | - | |
|  | Ill-defined or unspecified | 4,214 (4.6%) | 17 (4.4%) | | - | |
|  | Lip, oral cavity, pharynx | 3,557 (3.9%) | 17 (4.4%) | | - | |
|  | Lymphoid and haematopoietic tissue | 8,273 (9.1%) | 92 (24.0%) | | - | |
|  | Male genital organs | 10,896 (11.9%) | 22 (5.7%) | | - | |
|  | Malignant melanoma | 3,455 (3.8%) | ≤ 5 | | - | |
|  | Mesothelial and soft tissue | 1,886 (2.1%) | 17 (4.4%) | | - | |
|  | Respiratory and intrathoracic organs | 8,230 (9.0%) | 34 (8.9%) | | - | |
|  | Skin other than melanoma | 8,507 (9.3%) | 18 (4.7%) | | - | |
|  | Thyroid and other endocrine glands | 3,621 (4.0%) | ≤ 5 | | - | |
|  | Urinary tract | 6,059 (6.6%) | 13 (3.4%) | | - | |
| **Total** | Bone and articular cartilage | 913 (0.6%) | ≤ 5 | | - | |
|  | Breast | 12,555 (8.0%) | 20 (2.2%) | | - | |
|  | Digestive organs | 24,911 (15.9%) | 208 (22.9%) | | - | |
|  | Eye, brain and other parts of CNS | 9,115 (5.8%) | 22 (2.4%) | | - | |
|  | Female genital organs | 9,858 (6.3%) | 62 (6.8%) | | - | |
|  | Ill-defined or unspecified | 6,509 (4.1%) | 48 (5.3%) | | - | |
|  | Lip, oral cavity, pharynx | 6,940 (4.4%) | 43 (4.7%) | | - | |
|  | Lymphoid and haematopoietic tissue | 15,273 (9.7%) | 247 (27.2%) | | - | |
|  | Male genital organs | 16,142 (10.3%) | 35 (3.9%) | | - | |
|  | Malignant melanoma | 5,703 (3.6%) | 7 (0.8%) | | - | |
|  | Mesothelial and soft tissue | 3,192 (2.0%) | 49 (5.4%) | | - | |
|  | Respiratory and intrathoracic organs | 13,934 (8.9%) | 76 (8.4%) | | - | |
|  | Skin other than melanoma | 14,442 (9.2%) | 43 (4.7%) | | - | |
|  | Thyroid and other endocrine glands | 6,752 (4.3%) | ≤ 5 | | - | |
|  | Urinary tract | 10,688 (6.8%) | 41 (4.5%) | | - | |

**Table S10.** Temporally stratified results of the comparison between PLWH with cancer and the corresponding cancer patients without HIV.

| **Characteristics** | | **Cancer+/HIV- n (%)** | **Cancer+/HIV+ n (%)** | **P-Value (Fisher's exact test)** |
| --- | --- | --- | --- | --- |
| **Number of patients** | |  |  |  |
| 2005 - 2008 |  | 153 | 154 |  |
| 2009 - 2014 |  | 350 | 370 |  |
| 2015 - 2022 |  | 404 | 383 |  |
| Total |  | 907 | 907 |  |
| **Age at main cancer diagnosis** | |  |  |  |
| 2005 - 2008 | 18 - 39 years old | 42 (27.5%) | 46 (29.9%) |  |
|  | 40 - 59 years old | 68 (44.4%) | 83 (53.9%) |  |
|  | 60 - 79 years old | 40 (26.1%) | 24 (15.6%) |  |
|  | > 80 years old | ≤ 5 | ≤ 5 |  |
| 2009 - 2014 | 18 - 39 years old | 94 (26.9%) | 53 (14.3%) |  |
|  | 40 - 59 years old | 149 (42.6%) | 234 (63.2%) |  |
|  | 60 - 79 years old | 100 (28.6%) | 83 (22.4%) |  |
|  | > 80 years old | 7 (2.0%) | ≤ 5 |  |
| 2015 - 2022 | 18 - 39 years old | 104 (25.7%) | 54 (14.1%) |  |
|  | 40 - 59 years old | 163 (40.3%) | 218 (56.9%) |  |
|  | 60 - 79 years old | 127 (31.4%) | 106 (27.7%) |  |
|  | > 80 years old | 10 (2.5%) | ≤ 5 |  |
| Total | 18 - 39 years old | 240 (26.5%) | 153 (16.9%) |  |
|  | 40 - 59 years old | 380 (41.9%) | 535 (59.0%) |  |
|  | 60 - 79 years old | 267 (29.4%) | 213 (23.5%) |  |
|  | > 80 years old | 20 (2.2%) | 6 (0.7%) |  |
| **Metastasis occurence** | |  |  |  |
| 2005 - 2008 | N | 153 | 154 |  |
|  | Metastasis documented | 44 (28.8%) | 37 (24.0%) | *P* = .37 |
|  | Metastasis at time of cancer diagn. | 29 (65.9%) | 14 (37.8%) | *P* = .01 |
|  | Metastasis after cancer diagnosis | 15 (34.1%) | 23 (62.2%) | *P* = .01 |
| 2009 - 2014 | N | 350 | 370 |  |
|  | Metastasis documented | 126 (36.0%) | 118 (31.9%) | *P* = .27 |
|  | Metastasis at time of cancer diagn. | 76 (60.3%) | 58 (49.2%) | *P* = .09 |
|  | Metastasis after cancer diagnosis | 50 (39.7%) | 60 (50.8%) | *P* = .09 |
| 2015 - 2022 | N | 404 | 383 |  |
|  | Metastasis documented | 117 (29.0%) | 112 (29.2%) | *P* = .94 |
|  | Metastasis at time of cancer diagn. | 85 (72.6%) | 67 (59.8%) | *P* = .050 |
|  | Metastasis after cancer diagnosis | 32 (27.4%) | 45 (40.2%) | *P* = .050 |
| Total | N | 907 | 907 |  |
|  | Metastasis documented | 287 (31.6%) | 267 (29.4%) | *P* = .33 |
|  | Metastasis at time of cancer diagn. | 190 (66.2%) | 139 (52.1%) | *P* < .001 |
|  | Metastasis after cancer diagnosis | 97 (33.8%) | 128 (47.9%) | *P* < .001 |
| **Therapy modalities** | |  |  |  |
| 2005 - 2008 | N | 153 | 154 |  |
|  | Any major therapy documented | 95 (62.1%) | 103 (66.9%) | *P* = .41 |
|  | Surgery | 25 (16.3%) | 28 (18.2%) | *P* = .76 |
|  | Chemotherapy | 72 (47.1%) | 78 (50.6%) | *P* = .57 |
|  | Immunotherapy | 13 (8.5%) | 8 (5.2%) | *P* = .27 |
|  | Radiotherapy | 23 (15.0%) | 29 (18.8%) | *P* = .45 |
|  | Stem cell therapy | 17 (11.1%) | ≤ 5 | *P* = .003 |
|  | Bone marrow transplant | ≤ 5 | 0 | *P* = .12 |
|  | CAR T-cell therapy | 0 | 0 | *P* > 0.99 |
| 2009 - 2014 | N | 350 | 370 |  |
|  | Any major therapy documented | 257 (73.4%) | 275 (74.3%) | *P* = .80 |
|  | Surgery | 121 (34.6%) | 109 (29.5%) | *P* = .15 |
|  | Chemotherapy | 145 (41.4%) | 176 (47.6%) | *P* = .10 |
|  | Immunotherapy | 38 (10.9%) | 68 (18.4%) | *P* = .005 |
|  | Radiotherapy | 62 (17.7%) | 72 (19.5%) | *P* = .57 |
|  | Stem cell therapy | 33 (9.4%) | 14 (3.8%) | *P* = .002 |
|  | Bone marrow transplant | ≤ 5 | 0 | *P* = .11 |
|  | CAR T-cell therapy | 0 | 0 | *P* > 0.99 |
| 2015 - 2022 | N | 404 | 383 |  |
|  | Any major therapy documented | 279 (69.1%) | 261 (68.1%) | *P* = .82 |
|  | Surgery | 139 (34.4%) | 114 (29.8%) | *P* = .17 |
|  | Chemotherapy | 144 (35.6%) | 155 (40.5%) | *P* = .19 |
|  | Immunotherapy | 39 (9.7%) | 62 (16.2%) | *P* = .007 |
|  | Radiotherapy | 73 (18.1%) | 68 (17.8%) | *P* = .93 |
|  | Stem cell therapy | 34 (8.4%) | 15 (3.9%) | *P* = .01 |
|  | Bone marrow transplant | 6 (1.5%) | 0 | *P* = .03 |
|  | CAR T-cell therapy | ≤ 5 | 0 | *P* > 0.99 |
| Total | N | 907 | 907 |  |
|  | Any major therapy documented | 631 (69.6%) | 639 (70.5%) | *P* = .72 |
|  | Surgery | 285 (31.4%) | 251 (27.7%) | *P* = .09 |
|  | Chemotherapy | 361 (39.8%) | 409 (45.1%) | *P* = .03 |
|  | Immunotherapy | 90 (9.9%) | 138 (15.2%) | *P* < .001 |
|  | Radiotherapy | 158 (17.4%) | 169 (18.6%) | *P* = .54 |
|  | Stem cell therapy | 84 (9.3%) | 33 (3.6%) | *P* < .001 |
|  | Bone marrow transplant | 12 (1.3%) | 0 | *P* < .001 |
|  | CAR T-cell therapy | ≤ 5 | 0 | *P* > 0.99 |
| **Complications documented after chemotherapy** | |  |  |  |
| 2005 - 2008 | N | 153 | 154 |  |
|  | Chemotherapy documented | 72 (47.1%) | 78 (50.6%) | *P* = .57 |
|  | Complication after chemotherapy | 5 (6.9%) | 9 (11.5%) | *P* = .41 |
| 2009 - 2014 | N | 350 | 370 |  |
|  | Chemotherapy documented | 145 (41.4%) | 176 (47.6%) | *P* = .10 |
|  | Complication after chemotherapy | 7 (4.8%) | 35 (19.9%) | *P* < .001 |
| 2015 - 2022 | N | 404 | 383 |  |
|  | Chemotherapy documented | 144 (35.6%) | 155 (40.5%) | *P* = .19 |
|  | Complication after chemotherapy | 8 (5.6%) | 20 (12.9%) | *P* = .045 |
| Total | N | 907 | 907 |  |
|  | Chemotherapy documented | 361 (39.8%) | 409 (45.1%) | *P* = .03 |
|  | Complication after chemotherapy | 20 (5.5%) | 64 (15.6%) | *P* < .001 |

**Table S11.** Comparison of average length of stay and last documented discharge reason
stratified by year.

| **Characteristics** | | **Cancer+/HIV- n (%)** | **Cancer+/HIV+ n (%)** | **P-Value (Fisher's exact test)** |
| --- | --- | --- | --- | --- |
| **Average length of stay stratified by year of first relevant admission** | | |  |  |
| 2005 - 2008 | N | 153 | 225 |  |
|  | Up to 7 days on avg. | 55 (35.9%) | 65 (28.9%) | *P* = .18 |
|  | 7 - 14 days on avg. | 55 (35.9%) | 77 (34.2%) | *P* = .81 |
|  | 14 - 30 days on avg. | 31 (20.3%) | 68 (30.2%) | *P* = .04 |
|  | > 30 days on avg. | 12 (7.8%) | 15 (6.7%) | *P* = .82 |
| 2009 - 2014 | N | 261 | 299 |  |
|  | Up to 7 days on avg. | 112 (42.9%) | 101 (33.8%) | *P* = .03 |
|  | 7 - 14 days on avg. | 76 (29.1%) | 89 (29.8%) | *P* = .94 |
|  | 14 - 30 days on avg. | 48 (18.4%) | 78 (26.1%) | *P* = .04 |
|  | > 30 days on avg. | 25 (9.6%) | 31 (10.4%) | *P* = .87 |
| 2015 - 2022 | N | 300 | 190 |  |
|  | Up to 7 days on avg. | 152 (50.7%) | 62 (32.6%) | *P* < .001 |
|  | 7 - 14 days on avg. | 76 (25.3%) | 66 (34.7%) | *P* = .03 |
|  | 14 - 30 days on avg. | 54 (18.0%) | 52 (27.4%) | *P* = .02 |
|  | > 30 days on avg. | 18 (6.0%) | 10 (5.3%) | *P* = .89 |
| Total | N | 714 | 714 |  |
|  | Up to 7 days on avg. | 319 (44.7%) | 228 (31.9%) | *P* < .001 |
|  | 7 - 14 days on avg. | 207 (29.0%) | 232 (32.5%) | *P* = .17 |
|  | 14 - 30 days on avg. | 133 (18.6%) | 198 (27.7%) | *P* < .001 |
|  | > 30 days on avg. | 55 (7.7%) | 56 (7.8%) | *P* > 0.99 |
| **Last documented discharge categories stratified by year of last discharge** | | |  |  |
| 2005 - 2008 | N | 115 | 94 |  |
|  | Home | 88 (76.5%) | 60 (63.8%) | *P* = .06 |
|  | Deceased | 22 (19.1%) | 26 (27.7%) | *P* = .20 |
|  | Other Hospital | ≤ 5 | 0 | *P* < .001 |
|  | Rehabilitation or Residential Care | 0 | ≤ 5 | *P* < .001 |
|  | Hospice Care | ≤ 5 | ≤ 5 | *P* = .50 |
| 2009 - 2014 | N | 222 | 214 |  |
|  | Home | 178 (80.2%) | 135 (63.1%) | *P* < .001 |
|  | Deceased | 26 (11.7%) | 58 (27.1%) | *P* < .001 |
|  | Other Hospital | 13 (5.9%) | 9 (4.2%) | *P* = .57 |
|  | Rehabilitation or Residential Care | ≤ 5 | 8 (3.7%) | *P* = .53 |
|  | Hospice Care | 0 | ≤ 5 | *P* < .001 |
| 2015 - 2022 | N | 377 | 405 |  |
|  | Home | 317 (84.1%) | 321 (79.3%) | *P* = .10 |
|  | Deceased | 42 (11.1%) | 51 (12.6%) | *P* = .61 |
|  | Other Hospital | 8 (2.1%) | 19 (4.7%) | *P* = .08 |
|  | Rehabilitation or Residential Care | 6 (1.6%) | 8 (2.0%) | *P* = .89 |
|  | Hospice Care | ≤ 5 | 6 (1.5%) | *P* = .84 |
| Total | N | 714 | 713 |  |
|  | Home | 583 (81.7%) | 516 (72.4%) | *P* < .001 |
|  | Deceased | 90 (12.6%) | 135 (18.9%) | *P* = .001 |
|  | Other Hospital | 24 (3.4%) | 28 (3.9%) | *P* = .67 |
|  | Rehabilitation or Residential Care | 11 (1.5%) | 20 (2.8%) | *P* = .15 |
|  | Hospice Care | 6 (0.8%) | 14 (2.0%) | *P* = .11 |

**Figure S1.** Patient catchment area displaying proportions by patient group and site.

*Figure Notes*. Visualization of the patients’ catchment areas (Figure A1) illustrates the annual distribution of patients in each group (Cancer+/HIV+, Cancer+/HIV-, and Cancer-/HIV+) and by site. Across all sites, the largest catchment area was observed for the Cancer+/HIV- group, followed by the Cancer-/HIV+ group, whereas the Cancer+/HIV+ group had the smallest catchment area.

Travel distances to the hospital were analyzed across all centers, comparing distances among the three patient subgroups. Cancer+/HIV- patients had a mean travel distance of 25.9 km (95% CI, 25.6 to 26.3), whereas Cancer+/HIV+ patients traveled an average of 25.8 km (95% CI, 22.2 to 29.3), but with greater variability, with standard deviation ranging from 3.68 km to 520 km. Cancer-/HIV+ patients had the longest average travel distance at 30.4 km (95% CI, 28.2 to 32.5).

The Kruskal-Wallis test confirmed significant differences in travel distances among patient subgroups (*P* < .001), and Dunn’s post hoc test identified the greatest difference between Cancer-/HIV+ and Cancer+/HIV- patients (*Z* = -35.60; *P* < .001).


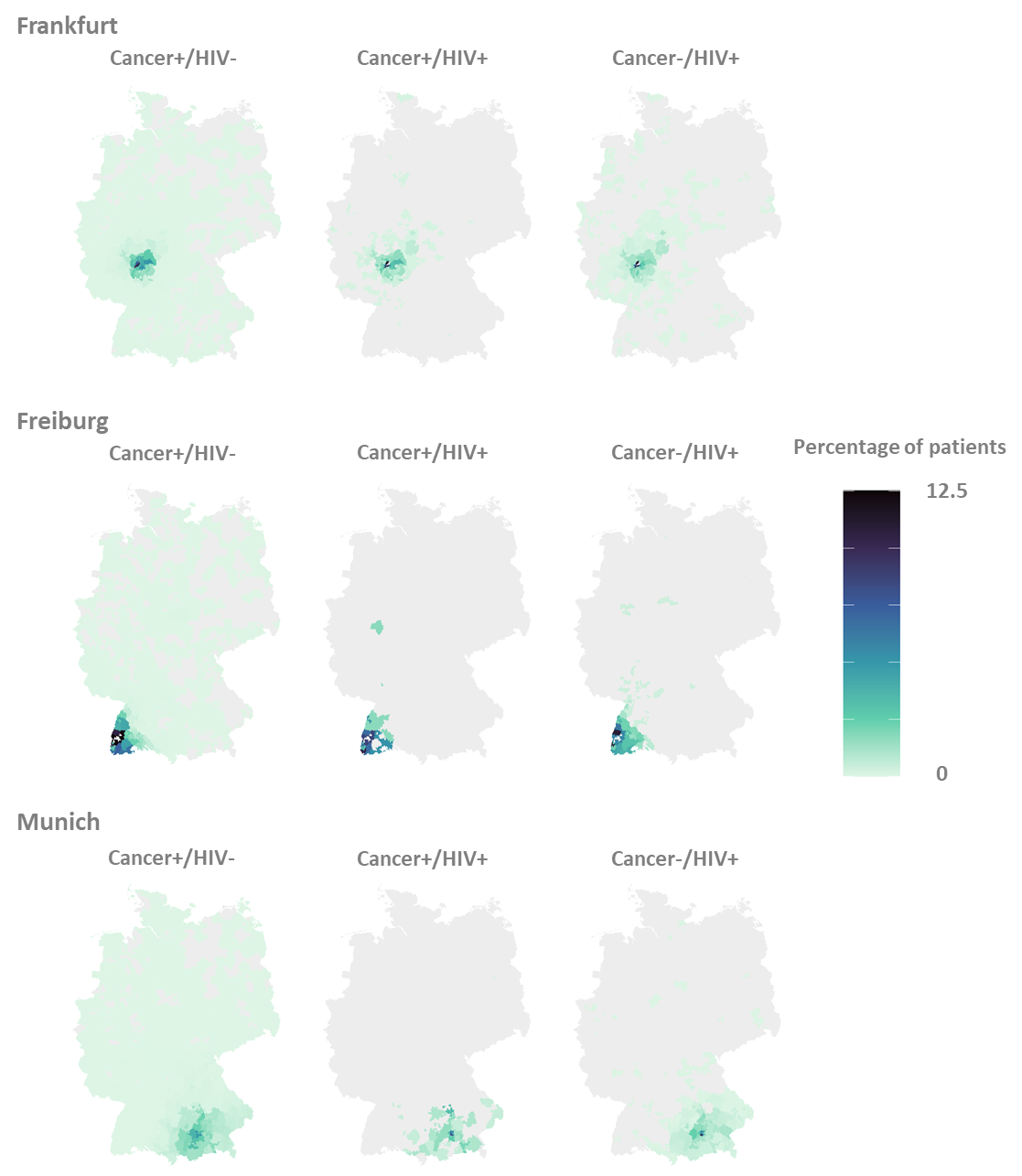

Supplement: Multimedia Appendix 1 [file publichealth-v12-e81092-s001.doc]
