# Supplementary figures and images for: Clinical Epidemiology of Cancer in People Living With HIV in Germany: Retrospective, Observational, Multicenter Federated Claims Data Analysis
Source: JMIR Public Health Surveill. 2026 Feb 13;12:e81092. doi: 10.2196/81092 (PMC12904353; doi:10.2196/81092)

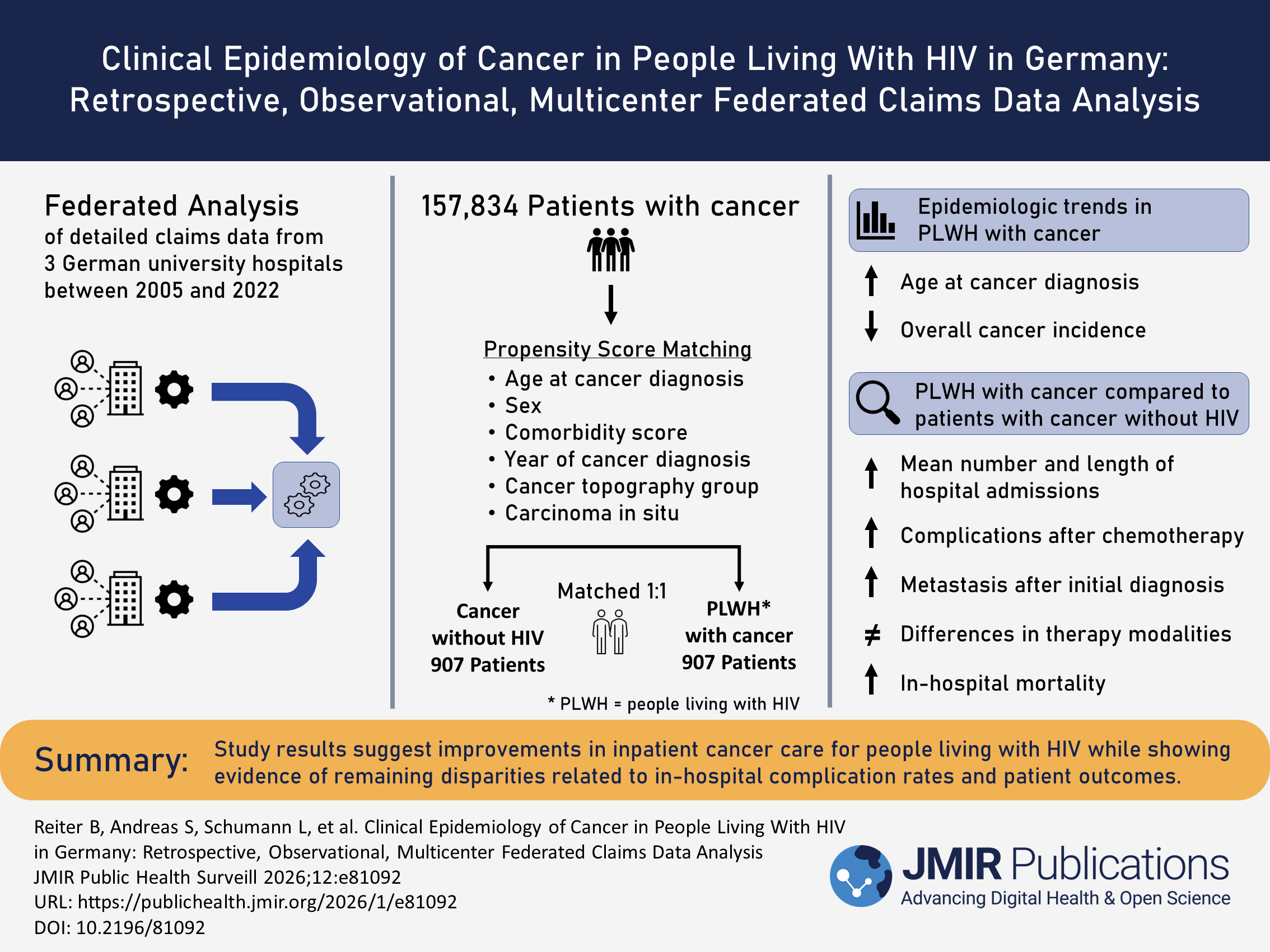

Supplement: Multimedia Appendix 2 [file publichealth-v12-e81092-s002.png]
